# Mesenchymal stem cells for inducing tolerance in organ transplantation

**DOI:** 10.3389/fcell.2014.00008

**Published:** 2014-03-17

**Authors:** Kequan Guo, Susumu Ikehara, Xu Meng

**Affiliations:** ^1^Department of Cardiac Surgery, Beijing Institute of Heart Lung and Blood Vessel Disease, Beijing Anzhen Hospital affiliated to Capital Medical UniversityBeijing, China; ^2^Department of Stem Cell Disorders, Kansai Medical UniversityHirakata City, Japan

**Keywords:** tolerance, mesenchymal stem cells, organ transplantation, mixed chimerism, bone marrow transplantation

## Abstract

Organ transplantation is useful for treating the end stage of organ failure. The induction of tolerance to the transplanted organ is essential for its long-term survival. Immunologic tolerance can be induced by immunosuppressive agents and mixed chimerism. Mixed chimerism is a state in which both recipient-and donor-derived blood cells remain in the hematopoietic system after allogeneic hematopoietic stem cells have been transplanted. Mesenchymal stem cells (MSCs), and immune cells such as dendritic cells and T-reg cells play an important role in the induction of tolerance. MSCs secrete cytokines, which modulate the immune response. In particular, they upregulate T-reg cell function and thereby induce tolerance. Intra-bone marrow-bone marrow transplantation recruits both donor-derived HSCs and MSCs, inducing persistent donor-specific tolerance without the use of immunosuppressants. In this review, we summarize the use of MSCs to induce tolerance in organ transplantation.

## Introduction

Organ transplantation is useful for treating the end stage of organ failure, but it is imperative that stable tolerance is induced in the recipients in order for the transplanted organ to survive long term. The induction of immunological tolerance to organ transplants can be categorized into the use of immunosuppressive agents to modulate T cell and B cell agents and the use of cellular therapies such as mixed chimerism (Salisbury et al., 2013). Immunosuppressive agents have been used to prevent an immune response in the host and the acute rejection of the transplanted organ. However, the side effects of non-specific immunosuppressive agents include not only the risk of infection but also the inability to prevent the chronic rejection of the transplanted organ (Ildstad et al., 2011). Mixed chimerism, which can be established by the transplantation of hematopoietic stem cells (HSCs), has been shown to be a better method of inducing tolerance in organ transplantation. In clinical practice, organ transplantation using a conditioning regimen consisting of total lymphoid irradiation plus immunosuppressants such as cyclophosphamide (CYP) and fludarabine has been shown to induce mixed chimerism (Sachs et al., 2014). One case report describes how a patient who received CD34+ cells (8 × 10^6^/kg body weight) on day14 after kidney transplantation, showed neither kidney rejection nor clinical manifestations of graft-versus-host disease (GVHD) during a follow-up for of more than 2 years (Scandling et al., 2008). Another review summarizes the induction of long-term allograft tolerance through mixed chimerism in small and large animal models, and includes clinical studies (Sachs et al., 2011). Allogeneic chimerism and tolerance have been induced by HSC transplantation in human leukocyte antigen-mismatched patients when kidneys were transplanted—without any evidence of acute GVHD in the recipients. And stable renal function was maintained as a result of the persistent donor chimerism without the use of immunosuppressive agents (Leventhal et al., 2012). Immune cells such as regulatory T (T-reg) cells, immature dendritic cells (DCs), and mesenchymal stem cells (MSCs) play important roles in the induction of immune tolerance in organ transplantation (Wood et al., 2012). Recent reports have suggested that MSC-derived-exosomes, which are released by exocytosis with the plasma membrane, may benefit therapy-refractory GvHD patients. The use of MSC exosomes, being-the therapeutically active component of MSCs, provides a number of advantages compared to just MSCs (Kordelas et al., 2014).

## Immune tolerance

Immune tolerance includes central tolerance, which occurs in the thymus, and peripheral tolerance, which includes the deletion of effector T cells and the induction of expansion of active T-reg cells (Wood and Sakaguchi, 2003). In central tolerance, T cell precursors from the bone marrow (BM) enter the thymus and are selected by thymic epithelial cells via positive and negative selection. These selected T cells show tolerance to autoantigens when they enter peripheral lymphoid tissues. Peripheral tolerance includes the induction of anergy, and the active regulation of effector T cells. Immature DCs, T reg cells and MSCs play an important role in the induction of peripheral tolerance. BM-derived immature DCs express low MHC class II and co-stimulatory molecules to promote tolerance to solid organ allografts, and the injection of donor-derived DCs may prevent the rejection of MHC-mismatched skin grafts (Roelen et al., 2003; van Kooten et al., 2011).

The markers of T-reg cells, CD4, CD25, and FoxP3, mainly mature in the thymus. The presence of T-reg cells has been reported to reduce the need for conditioning regimens in the generation of mixed chimerism (Raimondi et al., 2010), being the presence of both donor- and recipient-derived hematopoietic cells in the recipients after BM transplantation (BMT) (Pilat and Wekerle, 2010). It has been shown that mixed chimerism is more effective than full chimerism in combating infectious risk when allogeneic kidney transplants were performed in humans, with non-myeloablative conditioning promoting the mixed chimerism and helping the renal allografts to survive (Buhler et al., 2002; Kawai et al., 2011). MSCs can be isolated from many tissues, and not only support the growth of hematopoietic stem cells (HSCs), but also secrete cytokines to regulate the immune response. Human MSCs promote the generation of CD4+CD25+FoxP3+T-reg cells, and induce tolerance to allografts (Casiraghi et al., 2008; English et al., 2009). Moreover, MSCs secrete matrix metalloproteinases (MMP), protect allogeneic islets, and maintain long-term normoglycemia through MMP-2 and -9 *in vitro* (Ding et al., 2009).

## MSCs induce tolerance in organ transplantation

The functions of MSCs and their effects on immune cells have been summarized in two reviews (Uccelli et al., 2008; Li and Ikehara, 2013). MSCs suppress allogeneic T cell responses by secreting soluble factors such as PGE2, IL-10, and IL-6 (English, 2013), and modulate DC function, indirectly regulate T and B cell activity, delay and prevent the development of GVHD and suppress DC function (Zhang et al., 2009; Aldinucci et al., 2010). MSCs have been shown to alter the NK cell phenotype and suppress proliferation, decrease cytokine levels such as those of TNFα, IFNγ and IL-12, and increase IL-10. One report has shown that porcine MSCs inhibit alloreactive T cells through the induction of PGE2 and indoleamine 2,3-dioxygenase (IDO) (Hsu et al., 2013).

The immunoregulatory properties of MSCs have been reported *in vitro* and *in vivo*. MSCs suppress T-cell responses, inducing tolerance to transplanted kidney via the expression of IDO (Ge et al., 2010). An infusion of MSCs and rapamycin has been shown to induce heart allograft-specific tolerance, supporting the idea that MSCs might be used for inducing tolerance in a clinical setting (Ge et al., 2009). The MSC infusion leads to an expansion of T-reg cells and prolongs allograft survival in a MHC matched heterotopic heart transplantation model. Moreover, MSC infusion is characterized by reduced numbers of Th1 effector cells (Casiraghi et al., 2008). However, there is one report indicating that donor-specific MSC pre-treatment resulted in a higher degree of kidney cortex tissue damage and elevated creatinine levels in a rat kidney transplantation model (Seifert et al., 2012). Another report showed that MSCs suppressed allogeneic T-cell responses and prolonged the survival of transplanted hearts by improving the Th1/Th2 balance when allogeneic heart transplantation was combined with the intravenous infusion of MSCs (Zhou et al., 2006). Transplanted MSCs may promote revascularization and improve islet graft function after the co-transplantation of islets with MSCs in streptozotocin-induced diabetic rats (Ito et al., 2010).

In clinical trials, intravenous infusions of autologous BM-derived MSCs were given to kidney allograft recipients. Although immunosuppression remained unaltered, there was a resolution of tubulitis without interstitial fibrosis/tubular atrophy in one third of patients. Additionally, five of the six patients displayed a donor-specific downregulation of peripheral blood mononuclear cell proliferation, which was not reported in patients that did not have the MSC treatment. These results suggest that autologous BM-derived MSC treatment provides systemic immunosuppression in allograft transplantation (Reinders et al., 2013). Calcineurin inhibitors (CNIs) have been reported to reduce acute rejection rates in kidney recipients. The infusion of MSCs combined with CNIs improved renal function such as the estimated glomerular filtration rate at the first month after treatment. This therapy decreased the incidence of acute rejection, and also decreased the risk of opportunistic infection at 1 year after treatment (Tan et al., 2012). Infusion of MSCs was used for the treatment of patients who received kidney transplants, and these infused MSCs increased the percentage of CD4+CD25+FoxP3+CD127- T-reg cells and decreased memory T cells, and CD8+ T cell activity. The infusion of MSCs thus appears to be a safe and clinically feasible method for patients receiving organ transplants (Perico et al., 2011). BM-derived MSCs have been used clinically to treat GVHD, decrease the risk of infection, and help induce tolerance in organ transplantation, and one review indicates that MSCs promote tolerance in the case of kidney transplants (Casiraghi et al., 2014).

When MSCs are infused by intravenous injection, they become trapped in the lungs and other tissues, and it is therefore preferable for the MSCs to be directly injected into the bone cavity. Intra-bone marrow-BMT (IBM-BMT) has been shown to efficiently recruit not only donor-derived HSCs but also MSCs in animal experiments (Fukui et al., 2007; Guo et al., 2008; Song et al., 2008). Furthermore, IBM-BMT induced tolerance to adult allogeneic liver in mice (Okazaki et al., 2008). IBM-BMT is a feasible strategy for the induction of persistent donor-specific tolerance, enables the use of reduced radiation doses as conditioning regimens, and obviates the need for immunosuppressants (Guo et al., 2008). IBM-BMT induced tolerance in the case of allogeneic lung transplants, while intravenous BMT failed to do so (Kaneda et al., 2005). HSCs can normally proliferate in major histocompatibility complex (MHC)-compatible MSCs even in allogeneic microenvironments.

**Figure 1:**
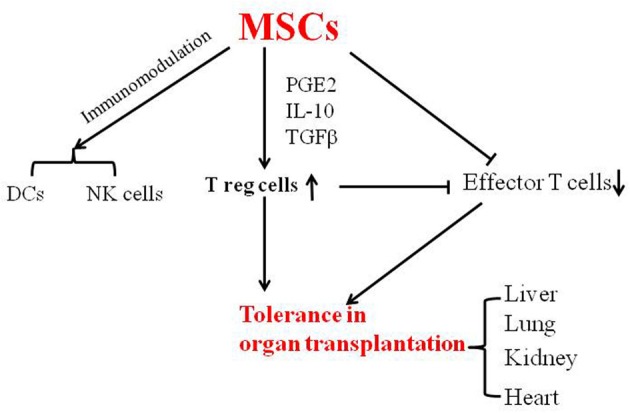
**MSCs induce tolerance in organ transplantation**.

In conclusion, MSCs have been shown to prevent GVHD, and to induce tolerance in organ transplantation in both animal and clinical studies (Figure 1). MSCs can be easily isolated from bone marrow and adipose tissue, and their use thus represents a feasible approach in the clinical setting for inducing tolerance in organ transplantation.

### Conflict of interest statement

The authors declare that the research was conducted in the absence of any commercial or financial relationships that could be construed as a potential conflict of interest.

## References

[B1] Aldinucci A. Rizzetto L. Pieri L. Nosi D. Romagnoli P. Biagioli T.. (2010). Inhibition of immune synapse by altered dendritic cell actin distribution: a new pathway of mesenchymal stem cell immune regulation. J. Immunol. 185, 5102–5110. 10.4049/jimmunol.1001332 20889545

[B2] Buhler L. H. Spitzer T. R. Sykes M. Sachs D. H. Delmonico F. L. Tolkoff-Rubin N.. (2002). Induction of kidney allograft tolerance after transient lymphohematopoietic chimerism in patients with multiple myeloma and end-stage renal disease. Transplantation 74, 1405–1409. 10.1097/00007890-200211270-00011 12451240

[B3] Casiraghi F. Azzollini N. Cassis P. Imberti B. Morigi M. Cugini D.. (2008). Pretransplant infusion of mesenchymal stem cells prolongs the survival of a semiallogeneic heart transplant through the generation of regulatory T cells. J. Immunol. 181, 3933–3946. 18768848 10.4049/jimmunol.181.6.3933

[B4] Casiraghi F. Remuzzi G. Perico N. (2014). Mesenchymal stromal cells to promote kidney transplantation tolerance. Curr. Opin. Organ Transplant. 19, 47–53. 10.1097/MOT.0000000000000035 24257324

[B5] Ding Y. Xu D. Feng G. Bushell A. Muschel R. J. Wood K. J. (2009). Mesenchymal stem cells prevent the rejection of fully allogenic islet grafts by the immunosuppressive activity of matrix metalloproteinase-2 and -9. Diabetes 58, 1797–1806. 10.2337/db09-0317 19509016 PMC2712800

[B6] English K. (2013). Mechanisms of mesenchymal stromal cell immunomodulation. Immunol. Cell Biol. 91, 19–26. 10.1038/icb.2012.56 23090487

[B7] English K. Ryan J. M. Tobin L. Murphy M. J. Barry F. P. Mahon B. P. (2009). Cell contact, prostaglandin E(2) and transforming growth factor beta 1 play non-redundant roles in human mesenchymal stem cell induction of CD4+CD25(High) forkhead box P3+ regulatory T cells. Clin. Exp. Immunol. 156, 149–160. 10.1111/j.1365-2249.2009.03874.x 19210524 PMC2673753

[B8] Fukui J. Inaba M. Ueda Y. Miyake T. Hosaka N. Kwon A. H.. (2007). Prevention of graft-versus-host disease by intra-bone marrow injection of donor T cells. Stem Cells 25, 1595–1601. 10.1634/stemcells.2006-0234 17446564

[B9] Ge W. Jiang J. Arp J. Liu W. Garcia B. Wang H. (2010). Regulatory T-cell generation and kidney allograft tolerance induced by mesenchymal stem cells associated with indoleamine 2,3-dioxygenase expression. Transplantation 90, 1312–1320. 10.1097/TP.0b013e3181fed001 21042238

[B10] Ge W. Jiang J. Baroja M. L. Arp J. Zassoko R. Liu W.. (2009). Infusion of mesenchymal stem cells and rapamycin synergize to attenuate alloimmune responses and promote cardiac allograft tolerance. Am. J. Transplant. 9, 1760–1772. 10.1111/j.1600-6143.2009.02721.x 19563344

[B11] Guo K. Inaba M. Li M. An J. Cui W. Song C.. (2008). Long-term donor-specific tolerance in rat cardiac allografts by intrabone marrow injection of donor bone marrow cells. Transplantation 85, 93–101. 10.1097/01.tp.0000296061.71662.76 18192918

[B12] Hsu W. T. Lin C. H. Chiang B. L. Jui H. Y. Wu K. K. Lee C. M. (2013). Prostaglandin E2 potentiates mesenchymal stem cell-induced IL-10+IFN-gamma+CD4+ regulatory T cells to control transplant arteriosclerosis. J. Immunol. 190, 2372–2380. 10.4049/jimmunol.1202996 23359497

[B13] Ildstad S. T. Shirwan H. Leventhal J. (2011). Is durable macrochimerism key to achieving clinical transplantation tolerance? Curr. Opin. Organ Transplant. 16, 343–344. 10.1097/MOT.0b013e328348e67a 21681096

[B14] Ito T. Itakura S. Todorov I. Rawson J. Asari S. Shintaku J.. (2010). Mesenchymal stem cell and islet co-transplantation promotes graft revascularization and function. Transplantation 89, 1438–1445. 10.1097/TP.0b013e3181db09c4 20568673

[B15] Kaneda H. Adachi Y. Saito Y. Ikebukuro K. Machida H. Suzuki Y.. (2005). Long-term observation after simultaneous lung and intra-bone marrow-bone marrow transplantation. J. Heart Lung Transplant. 24, 1415–1423. 10.1016/j.healun.2004.08.015 16143265

[B16] Kawai T. Cosimi A. B. Sachs D. H. (2011). Preclinical and clinical studies on the induction of renal allograft tolerance through transient mixed chimerism. Curr. Opin. Organ Transplant. 16, 366–371. 10.1097/MOT.0b013e3283484b2c 21666482 PMC3151013

[B17] Kordelas L. Rebmann V. Ludwig A. K. Radtke S. Ruesing J. Doeppner T. R.. (2014). MSC-derived exosomes: a novel tool to treat therapy-refractory graft-versus-host disease. Leukemia. . [Epub ahead of print]. 10.1038/leu.2014.41 24445866

[B18] Leventhal J. Abecassis M. Miller J. Gallon L. Ravindra K. Tollerud D. J.. (2012). Chimerism and tolerance without GVHD or engraftment syndrome in HLA-mismatched combined kidney and hematopoietic stem cell transplantation. Sci. Transl. Med. 4, 124ra28. 10.1126/scitranslmed.3003509 22399264 PMC3610325

[B19] Li M. Ikehara S. (2013). Bone-marrow-derived mesenchymal stem cells for organ repair. Stem Cells Int. 2013, 132642. 10.1155/2013/132642 23554816 PMC3608346

[B20] Okazaki S. Hisha H. Mizokami T. Takaki T. Wang X. Song C.. (2008). Successful acceptance of adult liver allografts by intra-bone marrow-bone marrow transplantation. Stem Cells Dev. 17, 629–639. 10.1089/scd.2007.0218 18537462

[B21] Perico N. Casiraghi F. Introna M. Gotti E. Todeschini M. Cavinato R. A.. (2011). Autologous mesenchymal stromal cells and kidney transplantation: a pilot study of safety and clinical feasibility. Clin. J. Am. Soc. Nephrol. 6, 412–422. 10.2215/CJN.04950610 20930086 PMC3052234

[B22] Pilat N. Wekerle T. (2010). Transplantation tolerance through mixed chimerism. Nat. Rev. Nephrol. 6, 594–605. 10.1038/nrneph.2010.110 20808286

[B23] Raimondi G. Sumpter T. L. Matta B. M. Pillai M. Corbitt N. Vodovotz Y.. (2010). Mammalian target of rapamycin inhibition and alloantigen-specific regulatory T cells synergize to promote long-term graft survival in immunocompetent recipients. J. Immunol. 184, 624–636. 10.4049/jimmunol.0900936 20007530 PMC2923839

[B24] Reinders M. E. de Fijter J. W. Roelofs H. Bajema I. M. de Vries D. K. Schaapherder A. F.. (2013). Autologous bone marrow-derived mesenchymal stromal cells for the treatment of allograft rejection after renal transplantation: results of a phase I study. Stem Cells Transl. Med. 2, 107–111. 10.5966/sctm.2012-0114 23349326 PMC3659754

[B25] Roelen D. L. Schuurhuis D. H. van den Boogaardt D. E. Koekkoek K. van Miert P. P. van Schip J. J.. (2003). Prolongation of skin graft survival by modulation of the alloimmune response with alternatively activated dendritic cells. Transplantation 76, 1608–1615. 10.1097/01.TP.0000086340.30817.BA 14702533

[B26] Sachs D. H. Kawai T. Sykes M. (2014). Induction of tolerance through mixed chimerism. Cold Spring Harb. Perspect. Med. 4, a015529. 10.1101/cshperspect.a015529 24384815 PMC3869282

[B27] Sachs D. H. Sykes M. Kawai T. Cosimi A. B. (2011). Immuno-intervention for the induction of transplantation tolerance through mixed chimerism. Semin. Immunol. 23, 165–173. . [Epub ahead of print]. 10.1016/j.smim.2011.07.001 21839648 PMC3178004

[B28] Salisbury E. M. Game D. S. Lechler R. I. (2013). Transplantation tolerance. Pediatr. Nephrol. 10.1007/s00467-013-2659-5 24213880 PMC4212135

[B29] Scandling J. D. Busque S. Dejbakhsh-Jones S. Benike C. Millan M. T. Shizuru J. A.. (2008). Tolerance and chimerism after renal and hematopoietic-cell transplantation. N. Engl. J. Med. 358, 362–368. 10.1056/NEJMoa074191 18216356

[B30] Seifert M. Stolk M. Polenz D. Volk H. D. (2012). Detrimental effects of rat mesenchymal stromal cell pre-treatment in a model of acute kidney rejection. Front. Immunol. 3:202. 10.3389/fimmu.2012.00202 22826709 PMC3398550

[B31] Song C. Hisha H. Wang X. Li Q. Li M. Cui W.. (2008). Facilitation of hematopoietic recovery by bone grafts with intra-bone marrow-bone marrow transplantation. Immunobiology 213, 455–468. 10.1016/j.imbio.2007.10.015 18514748

[B32] Tan J. Wu W. Xu X. Liao L. Zheng F. Messinger S.. (2012). Induction therapy with autologous mesenchymal stem cells in living-related kidney transplants: a randomized controlled trial. JAMA 307, 1169–1177. 10.1001/jama.2012.316 22436957

[B33] Uccelli A. Moretta L. Pistoia V. (2008). Mesenchymal stem cells in health and disease. Nat. Rev. Immunol. 8, 726–736. 10.1038/nri2395 19172693

[B34] van Kooten C. Lombardi G. Gelderman K. A. Sagoo P. Buckland M. Lechler R.. (2011). Dendritic cells as a tool to induce transplantation tolerance: obstacles and opportunities. Transplantation 91, 2–7. 10.1097/TP.0b013e31820263b3 21452405

[B35] Wood K. J. Bushell A. Hester J. (2012). Regulatory immune cells in transplantation. Nat. Rev. Immunol. 12, 417–430. 10.1038/nri3227 22627860

[B36] Wood K. J. Sakaguchi S. (2003). Regulatory T cells in transplantation tolerance. Nat. Rev. Immunol. 3, 199–210. 10.1038/nri1027 12658268

[B37] Zhang B. Liu R. Shi D. Liu X. Chen Y. Dou X.. (2009). Mesenchymal stem cells induce mature dendritic cells into a novel Jagged-2-dependent regulatory dendritic cell population. Blood 113, 46–57. 10.1182/blood-2008-04-154138 18832657

[B38] Zhou H. P. Yi D. H. Yu S. Q. Sun G. C. Cui Q. Zhu H. L.. (2006). Administration of donor-derived mesenchymal stem cells can prolong the survival of rat cardiac allograft. Transplant. Proc. 38, 3046–3051. 10.1016/j.transproceed.2006.10.002 17112896

